# Canine Brucellosis: Insights Into the Epidemiologic Situation in Europe

**DOI:** 10.3389/fvets.2019.00151

**Published:** 2019-05-31

**Authors:** Gesine Buhmann, Frauke Paul, Werner Herbst, Falk Melzer, Georg Wolf, Katrin Hartmann, Andrea Fischer

**Affiliations:** ^1^Clinic of Small Animal Medicine, Centre for Clinical Veterinary Medicine, LMU Munich, Munich, Germany; ^2^IDEXX Laboratories, Ludwigsburg, Germany; ^3^Department of Veterinary Medicine, Institute of Hygiene and Infectious Diseases of Animals, University of Giessen, Gießen, Germany; ^4^Institute of Bacteriological Infections and Zoonosis, Friedrich-Loeffler-Institute, Federal Research Institute for Animal Health, Jena, Germany; ^5^Department of Veterinary Science, Institute for Infectious Diseases and Zoonoses, LMU Munich, Munich, Germany

**Keywords:** Canine brucellosis, *Brucella canis*, zoonosis, epidemiology, dog, European countries, discospondylitis

## Abstract

*Brucella canis* is one of many responsible pathogens of discospondylitis in dogs and infections require specific management. Little is known about the epidemiologic situation in Europe. The purpose of the study was to get insights into the occurrence of brucellosis in dogs in Europe. The database of a European veterinary laboratory was screened for *Brucella* positive samples. Additionally, medical records of a veterinary hospital in Germany were screened for diagnosis of discospondylitis and brucellosis. The laboratory received samples from 20 European countries for *Brucella* testing in dogs: 3.7% of submitted samples were *Brucella* spp. PCR-positive (61/1,657), and *Brucella canis* antibodies were identified in 5.4% of submitted samples (150/2,764). *Brucella* spp. PCR-positive samples originated from Spain (11.1% of submitted samples), Poland (6.7% of submitted samples) and rarely from Italy and France. Samples with *Brucella canis* antibodies originated from 13 European countries (Sweden, Belgium, Austria, Switzerland, Italy, Finland, Germany, Denmark, Hungary, Norway, Poland, France, Netherlands). Young dogs (0–24 months) had a 5.4-fold increased risk of PCR positive samples. The supplementary medical records search identified four young female dogs (7–30 months) with *Brucella canis* discospondylitis in Germany. The four dogs had been imported to Germany from Eastern European countries (Moldavia, Romania, Macedonia). In conclusion, infection with *Brucella canis* needs to be considered in dogs in Europe and diagnostics for *Brucella canis* infection appear indicated in young dogs with discospondylitis.

## Introduction

Veterinary neurologists are frequently confronted with spinal pain in dogs. One differential diagnosis of spinal pain is discospondylitis. The most common etiologies of discospondylitis in dogs are *Staphylococcus* species, and less frequently *Streptococcus* species, *Escherichia coli, Aspergillus* species and *Brucella canis* or *Brucella suis*, as well. Routine diagnosis relies on spinal radiographs, CT or MRI, blood cultures and needle aspirates of effected disc spaces using fluoroscopy or CT. A special approach is required for diagnosis of *Brucella* discospondylitis (1–3).

*Brucella canis* (*B. canis*) is a gram-negative, facultative intracellular coccobacillus which has been reported in many regions of the world and is considered endemic in Southern USA, in Central and South America and in Mexico (4–8). *B. canis* also occurs in Canada (9). Frequent reports of infections with *B. canis* also originate from Asia (China, Japan, India) and Africa (Nigeria, Zimbabwe) (5, 10–13). It is exotic in Australia and does not occur in New Zealand (14–16). In Germany, *B. canis* was reported in 1976 in a colony of Beagle dogs (17) and 2003 in one male dog with epididymitis and orchitis (18). Rare cases originate also from other European countries, such as Sweden (19, 20), the United Kingdom (21, 22), Austria (23), Italy (24), and Hungary (25). It is generally assumed that countries with a large stray dog population have a higher prevalence of infection, since stray dogs can contribute to distribution and retention of this organism in dog populations (6, 13, 26, 27). Complementary, breeding colonies are at increased risk of maintaining the infection, as well (28).

*B. canis* discospondylitis is the most frequently reported manifestation outside the genital tract (24, 29–33), others are generalized lymphadenopathy (19), intraocular inflammation (34, 35), and rarely osteomyelitis (36) and meningoencephalitis (37) with similar appearance as neurobrucellosis in humans (38). Common consequences of *B. canis* infection in dogs are late abortion, stillbirth, and failure to conceive in female dogs (8, 19, 39, 40) and epididymitis, orchitis, prostatitis and infertility in male dogs (24, 29, 41). Puppies can be born, which have very high risk of perinatal mortality (37). A major concern is that *B. canis* can cause a lifelong infection with intermittent shedding of bacteria (42, 43). Awareness of canine brucellosis as a zoonosis increased in the last years (7, 44, 45), although symptomatic human infections are considered rare (46). Transmission of *B. canis* from dogs to humans is possible. Immunocompromized people with close contact to infected dogs (44, 47, 48), and laboratory workers handling infected specimen are considered at risk for infection (46, 49–51). Yet, surveillance for brucellosis as a zoonotic disease commonly focuses on *B. melitensis, B. suis* and *B. abortus* (52–54). Less attention has been paid to *B. canis* in Europe, although dogs usually live in close contact with their owners.

The purpose of the study was to get insights into the occurrence of *Brucella canis* in dogs in Europe.

We screened the database of a European laboratory for *Brucella* positive samples. Additionally, medical records of a German veterinary hospital were reviewed for dogs with *Brucella canis* discospondylitis and their geographic origin.

## Materials and Methods

The database of a veterinary diagnostic laboratory (IDEXX laboratories, Ludwigsburg, Germany), which received samples from dogs for *B. canis* testing from 20 European countries, was investigated (2011–2016). The laboratory had received 4,421 samples from dogs for testing for *Brucella* infection: 1,657 samples were submitted for detection of *Brucella* spp. with polymerase chain reaction (PCR), and 2,764 samples were submitted for detection of *B. canis* antibodies. Four samples were marked as originating from the same two dogs (Supplementary Tables 1–3). Samples were submitted by veterinarians or dog breeders. Polymerase chain reaction was a real-time PCR (IDEXX RealPCR™), which amplified the 76 bp-sequence of the internal transcribed spacer gene region of *Brucella* spp. The IDEXX RealPCR™ detects *B. canis, B. microti, B. melitensis, B. abortus, B. suis*, and *B. ovis*. Sequencing was not performed. Antibody test was routinely performed with an agglutination test (Institute of Hygiene and Infectious Diseases of Animals, JLU Gießen, Gießen, Germany). *B. canis* strain RM 6/66 was used as antigen. After growth on tryptone soy bean agar plates in a 5% CO_2_ enriched atmosphere for 48 h at 37°C bacteria were harvested and suspended in 0.15 M phosphate buffered saline (PBS) (pH 7.2). The suspension was filtered through four layers of gauze and subsequently heated in a water bath at 56°C for 90 min to inactivate the microorganism. After washing the bacteria twice in PBS the last pellet was suspended in the same buffer to an about 10-fold higher concentration as required for the agglutination test. As a preservative Merthiolate was added (final concentration of 0.01%). For the agglutination test the concentrated antigen was diluted in 0.15 M NaCl giving a turbidity of McFarland no. 5. Each dilution (0.5 ml) of a log 2 dilution series in 0.15 M NaCl, beginning with 1:25, of the field sera was mixed with the same volume of the antigen suspension giving a final serum dilution of 1:50, and then incubated for 48 h at 37°C in a humidified atmosphere. The reciprocal value of the last dilution which still revealed an at least 50% agglutination of the *B. canis* cells was recorded as titer. For controls known negative (field serum) and positive dog sera were used. The latter was from a dog experimentally infected with *B. canis*. According to Carmichael and Greene a titer of 100 was chosen as cut off value (43, 55, 56).

The following data were retrieved from the laboratory database: Submitted materials for diagnostic testing for *B. canis* infection, requested diagnostic tests (*B. canis* antibodies, *Brucella* spp. PCR), diagnostic test results for *B. canis* antibodies (presence, absence), diagnostic test results for *Brucella* spp. PCR (positive, negative), country of origin of the samples, sex, and age of the dogs. Frequency of PCR-positive samples and frequency of samples with *B. canis* antibodies were calculated as percentages of all submitted samples and for each country. Confidence intervals (CI_95%_; calculation according to Abraham Wald) were calculated in excel (Microsoft Office Standard 2013) with the following formula: $CI_{95\%}=P\pm 1,96\sqrt{\frac{P\left(1-P\right)}{n}}$ [*n*: total number of samples tested; P: proportion of samples with positive test results (%)]. The strength of association between positive PCR or antibody presence and sex and age was estimated by calculation of odds ratios (ORs). An odds ratio with a 95% CI excluding 1 was considered to indicate a significant association at the 5% level.

As a secondary supplementary study, the medical records of a neurology service in a German veterinary hospital were reviewed for dogs with a diagnosis of discospondylitis and *Brucella canis* infection. Data on diagnostics, traveling history and geographic origin of the dogs were extracted (Supplementary Table 4).

The ethics committee of the veterinary faculty LMU Munich approved the study (114-16-02-2018).

## Results

**Figure 1:**
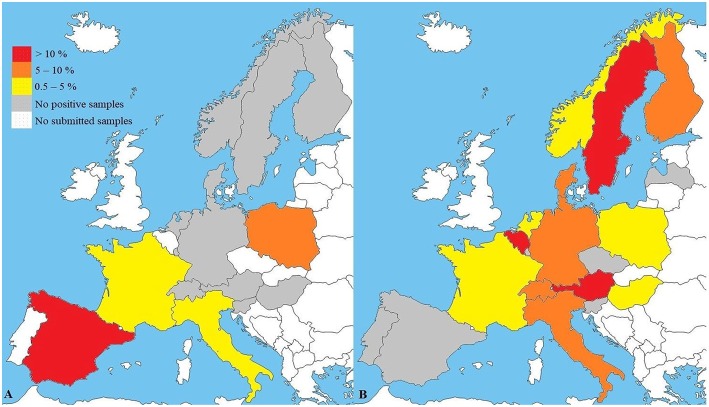
**(A**) Results of *Brucella* spp. PCR (*n* = 1,657) and **(B)** *B. canis* antibody testing (*n* = 2,764) in a veterinary diagnostic laboratory. Maps reflect preference to use PCR **(A)** or antibody tests **(B)** in respective countries. *B. suis* may not be recognized. Sample sizes and confidence intervals (CI_95%_) for each country are described in Tables 2, 3.

The European veterinary diagnostic laboratory had received 4,421 samples from 4,419 dogs for testing for canine brucellosis within a 5-year period (Table 1). Samples submitted to the laboratory for *Brucella* spp. PCR originated from 15 European countries. PCR was positive in 3.7% (61/1,657) of all submitted samples. *Brucella* spp. PCR-positive samples originated from four European countries: Spain, Poland, Italy and France (Figure 1A). Sample sizes for each country are described in Table 2. Young dogs (0–2 years old) had a 5.4-fold increased risk for being PCR-positive (OR 5.4; CI_95%_ 2.2–13.7) compared to dogs aged 3 years of age or older (OR 0.2; CI_95%_ 0.1–0.5). Statistical analysis failed to demonstrate an association between the sex of the dogs and positive *Brucella* spp. PCR. Samples submitted to the laboratory for *B. canis* antibody testing originated from 20 European countries. Presence of antibodies was documented in 5.4% (150/2,764) of all submitted samples. Samples with *B. canis* antibodies originated from 13 European countries: Sweden, Belgium, Austria, Switzerland, Italy, Finland, Germany, Denmark, Hungary, Norway, Poland, France and the Netherlands (Figure 1B). Sample sizes for each country are described in Table 3. Young dogs had a lower risk of being antibody-positive (OR 0.6; CI_95%_ 0.4–0.9). Conversely, an increased risk for *B. canis* antibody positivity was observed in dogs aged 3 years or older (OR 1.6; CI_95%_ 1.1–2.2). Statistical analysis failed to demonstrate an association between the sex of the dogs and the presence of *Brucella canis* antibodies.

**Table 1:** Submitted samples and diagnostic test results for *B. canis* in the laboratory (2011–2016).

	***Brucella*** **spp. PCR**		***B. canis*** **antibodies**	
	**Submitted samples** **(*n* = 1657)**	**Positive samples** **(*n* = 61)**	**Submitted samples** **(*n* = 2764)**	**Positive samples** (*n* = 150^*^)
**SEX**				
Female	600	2	938	58
Male	300	9	1188	61
Unknown	757	50	636	29
**AGE**				
0–2 years	1085	55	1449	63
3–5 years	383	4	595	45
6–8 years	104	0	348	21
>8 years	27	1	216	12
Unknown	58	1	154	7
**MATERIAL**				
Whole blood	47	0	279	20
Serum	1	1	2484	130
Urine	8	0	0	0
feces	1	0	0	0
Ejaculate	21	0	0	0
Cerebrospinal fluid	12	0	1	0
Synovial fluid	1	0	0	0
Bronchoalveolar lavage	1	0	0	0
Mucosal swab of genital tract	70	0	0	0
Mucosal swab of rectum	1	0	0	0
Unknown swabs/aspirates	1256	56	0	0
Tissue of testis	4	0	0	0
Tissue of aborted material	1	0	0	0
Tissue of skin	1	0	0	0
Unknown biopsies/tissues	231	4	0	0
Bone marrow	1	0	0	0

* *150 samples from 148 dogs*.

**Table 2:** Submitted samples for *Brucella* spp. PCR testing: Country of origin, positivity and confidence intervals (CI_95%_).

**Country of origin**	**Submitted samples**	**Positive samples**	**Positive%**	**CI_**95%**_**
Spain	253	28	11.10	7.20–14.90%
Poland	432	29	6.70	4.40–9.10%
Italy	103	1	1.00	0.00–2.90%
France	382	3	0.80	0.00–1.70%
Germany	386	0	0.00	–
Netherlands	32	0	0.00	–
Austria	35	0	0.00	–
Hungary	13	0	0.00	–
Denmark	9	0	0.00	–
Switzerland	5	0	0.00	–
Finland	2	0	0.00	–
Luxembourg	2	0	0.00	–
Sweden	1	0	0.00	–
Slovenia	1	0	0.00	–
Norway	1	0	0.00	–
All samples	1,657	61	3.70	2.80–4.60%

**Table 3:** Submitted samples for *B. canis* antibody testing: Country of origin, positivity and confidence intervals (CI_95%_).

**Country of origin**	**Submitted samples**	**Positive samples**	**Positive%**	**CI_**95%**_**
Sweden	22	3	13.60	0.00–28.00%
Belgium	49	6	12.20	3.10–21.40%
Austria	95	11	11.60	5.10–18.00%
Switzerland	85	7	8.20	2.40–14.10%
Italy	215	17	7.90	4.30–11.50%
Finland	203	14	6.90	3.40–10.40%
Germany	1065	58	5.40	4.10–6.80%
Denmark	117	6	5.10	1.10–9.10%
Hungary	140	6	4.30	0.90–7.60%
Norway	73	3	4.10	0.00–8.70%
Poland	164	6	3.70	0.80–6.50%
France	415	11	2.70	1.10–4.20%
Netherlands	98	2	2.00	0.00–4.80%
Czech Republic	8	0	0.00	–
Luxembourg	5	0	0.00	–
Malta	3	0	0.00	–
Latvia	3	0	0.00	–
Spain	2	0	0.00	–
Portugal	1	0	0.00	–
Slovenia	1	0	0.00	–
All samples	2,764	150	5.40	4.60–6.30%

**Figure 2:**
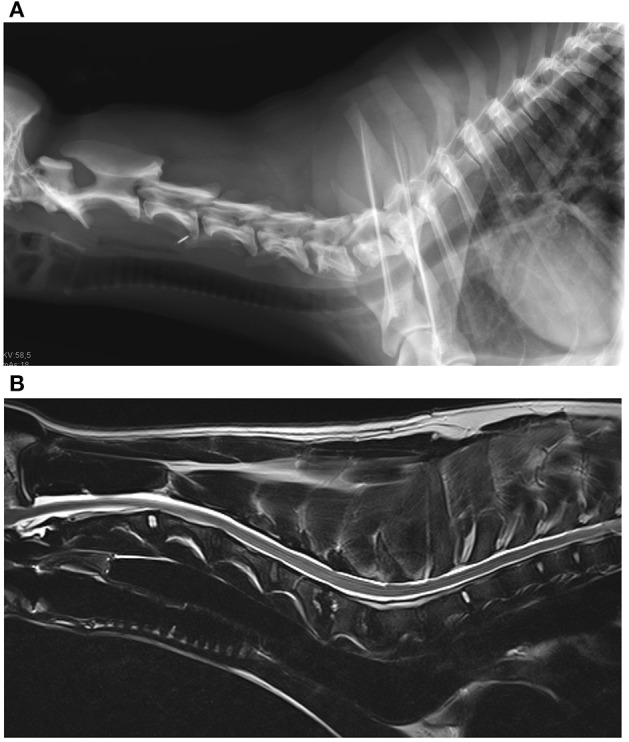
**(A)** Radiograph of the cervical spine of a dog with Brucella canis discospondylitis. There is lysis of the caudal vertebral end plate of C4 and the cranial end plate of C5 and bone production around the periphery of the vertebral bodies. **(B)** Magnetic resonance images of the same dog. Sagittal T2-weighted image of the cervical spine. The caudal end plate of C4 and the cranial end plate of C5 appear hyperintense to adjacent vertebral bodies, the intervertebral disc space appears narrowed. The caudal endplate of C5 and the cranial end plate of C6 also show similar lesions, but in a milder condition (Imaging: Clinic for Surgery and Reproduction in Small Animals, Veterinary Faculty, Ludwig-Maximilians-University, Munich, Germany).

The medical records search identified four dogs with *B. canis* infection and discospondylitis (Figures 2A,B). The four dogs (3 female-spayed, 1 female-intact; age 7–30 months) originated from Eastern European countries (1 Moldavia, 2 Romania, 1 North Macedonia) and had been brought to Germany between one and 23 months prior to presentation. None of the dogs had been used for breeding purposes. Diagnosis of brucellosis was based on growth on bacterial blood cultures and bacteria identification by MALDI-TOF mass spectrometry (3 dogs) or presence of *B. canis* antibodies [1 dog, tested twice, agglutination test and indirect fluorescent antibody test (IFAT 1:512)]. Two additional multiplex PCR assays (Bruce-ladder and New Bruce-ladder) confirmed growth of *B. canis* on blood cultures (3 dogs). Using these methods most of the known *Brucella* species can be identified by specific band patterns of amplificates of different sizes. It is even possible to differentiate between *B. suis* biovars and *B. canis* (57, 58).

## Discussion

This study provides insights into the regional occurrence of *B. canis* in dogs in Europe. The evaluation of the European laboratory's database showed that PCR- and antibody-positive samples originated from dogs from a variety of European countries, suggesting the widespread presence of *B. canis* in Europe. *Brucella* spp. DNA was present in 3.7% of all submitted samples and *B. canis* antibodies in 5.4% of samples submitted for diagnostic testing for canine *Brucella* infection from 20 European countries. Yet, the true occurrence of *B. canis* infection in dogs in Europe remains unknown. Data presented here reveal only occurrence in samples in which testing for *B. canis* was specifically requested by the veterinarian or originate from populations at risk and, thus, are not representative of the exact occurrence in the dog population of the respective country. Limitations of the present study are that the results refer to samples from preselected dogs and do not reflect countrywide occurrence. The samples were collected from a heterogeneous group of dogs, so the proportions of positive tested samples must be interpreted critically and cannot measure a real country prevalence of canine brucellosis. Another limitation in the interpretation of the data is the non-species-specific PCR used by the laboratory. Dogs are susceptible to infections with *B. abortus, B. suis* and *B. melitensis*. Infections with other *Brucella* spp. than *B. canis* should be considered in countries in which brucellosis is not yet eradicated, if the dog is fed raw pig meat or is a hunting dog or is housed on a farm (2, 16, 59). In addition, number of samples from several countries was insufficient for estimating an accurate frequency of positivity as reflected by the confidence intervals (Tables 2 and 3). In the present study, the laboratory database only specified the country of origin, but otherwise the precise origin of the samples remained unknown. Furthermore, the diagnosis of *B. canis* infection with antibodies faces several limitations: False positive results can can result from cross-reactions with other bacteria that offer the same antigenic determinants or tests can be negative early during infection in the first 3 to 8 weeks (39, 43, 53, 60). There are no official databases: Canine brucellosis (*B. canis)* is not notifiable to WHO or EU compared to brucellosis in cattle (*B. abortus*), pigs (*B. suis*), sheep and goats (*B. melitensis*). Furthermore, investigations on *B. canis* oftentimes are restricted to preselected groups of dogs from kennels with reproductive problems or single incidences of discospondylitis (20, 23, 25). Support for the findings of the present study comes from a number of recent reports of *B. canis* infection in dogs from several other European countries, such as Sweden (19, 20), the United Kingdom (21, 22), Austria (23), Italy (24), Hungary (25) or Switzerland (61).

Bacterial isolation has been considered the only proof of *B. canis* infection (39, 60, 62). More recently, PCR of clinical samples has been suggested as a diagnostic test for detection of subclinical and early infections prior to antibody development (63–67). PCR detects *Brucella* spp. DNA in samples, and positive results indicate infection with *Brucella* spp. Thus, results of samples from Spain and Poland suggest that *B. canis* occurs in these countries (20). In samples from Spain, 11.1% had positive PCR results, and in samples from Poland, 6.7% showed positive PCR results and 3.7% revealed the presence of *B. canis* antibodies. So far, there is a lack of reports on *B. canis* infections in Polish dogs. Iwaniak et al. (68) even stated that *B. canis* had never been confirmed in Poland (68). Yet, a case report from Sweden in 2012 established a connection between a bitch with *B. canis* infection that aborted repeatedly, and a stud dog from Poland (19).

Antibody testing was only available from few Spanish samples, which are not representative, but previously Mateu de Antonia et al. (69) indicated *B. canis* antibody prevalence for Spanish stray dogs as high as 6.5% (69). There was also a report on a brucellosis outbreak in a Swedish kennel, in which two diseased bitches had been mated with stud dogs from Spain (20). It may also be of interest that the present study revealed *B. canis* antibodies in 6.9% of samples from Finland, which is in contrast to data from Dalhbom et al. (70), who had shown that 388 dogs from 94 Finnish kennels had no *B. canis* antibodies (70). An important limitation of epidemiologic studies of *Brucella canis* based on antibodies is the fact that positive samples were not tested twice. In order to exclude false positive results, confirmation with a second serological test would be advantageous. Thus, further investigations are warranted to investigate regional differences in *B. canis* occurrence. The results of this study have also implications for the diagnosis of discospondylitis. The gold standard for diagnosis of the specific underlying etiology of discospondylitis is bacterial isolation with blood and urine cultures, or in case of negative blood/urine cultures with cultures of image-guided disc biopsies (1, 3). However, advantages of PCR are to detect acute or chronic infections and the possibility to examine blood, urine and secretions. Especially, vaginal swabs or semen are suitable materials for *Brucella* diagnosis with PCR (63, 71). In our study, swabs and aspirates of unknown origin were the most frequently submitted material with positive test results. Combination of different materials for PCR-testing can increase the diagnostic success (71). Consequently, routine testing with PCR from several specimens could enable rapid diagnosis of canine brucellosis in practice and may also be employed for future epidemiologic investigations. The laboratory data shows an increased risk (5.4-fold) of PCR-positive samples in young dogs up to 2 years of age and confirms the overrepresentation of young dogs in previous case reports (19, 22, 29, 35). Another study strengthens this consideration, here, 21% of 200 canine neonates had a positive *B. canis*-PCR (72). Adjacent to vertical transmissions between bitches and their puppies via placenta, during parturition or weaning, potential other modes of infection are venereal transmission in sexually mature dogs and infection via mucosal membranes of oropharynx or conjunctiva (urine, vaginal secretions) (37, 73). *B. canis* is well-known to cause abortions and stillbirth in infected bitches. However, puppies can be born and may either die shortly after birth or appear seemingly healthy and develop the disease later (19, 37). Thus, chronically infected surviving puppies could present a serious source for harbourage and spread of *B. canis* (25).

In general, treatment of *B. canis* diseased dogs is controversially discussed because of the risk of harboring this agent lifelong and its zoonotic potential (19, 39). Veterinary literature considers treatment of *B. canis* infection in dogs with combined use of tetracyclines and aminoglycosides (26, 29, 43). WHO guidelines recommend combined treatment with tetracycline or doxycycline and an aminoglycoside antibiotic or rifampicin in humans (74, 75). Combination of doxycycline and rifampicin seems to be effective in dogs infected with *B. suis*, as well (2). Treatment with enrofloxacin eliminated clinical signs and frequent abortions in a kennel with twelve infected breeding dogs (76). A new approach was the additional administration of hydroxychloroquine, which improved clinical signs at an early stage, and reduced treatment failures and relapses in human patients with brucellosis (77). This drug has already been used as an immunomodulatory drug in dogs with lupus erythematosus or lymphoma (78, 79). Indeed, no antibiotic therapy appears to eliminate *B. canis* completely from affected dogs, and treatment failures and relapses are frequently reported (19, 26, 39).

Literature considers the zoonotic potential of *B. canis* is low compared to *B. melitensis, B. suis* and *B. abortus*, which are more frequently reported as underlying cause of human brucellosis (52, 80–83). Less attention has been paid to *B. canis* in Europe, although dogs usually live in close contact with their owners and there is an increasing incidence of dog trafficking and import of puppies from breeding kennels with poor state of health (19, 84). An increasing number of case reports describes *B. canis* infections in people, especially in immunocompromized adults or children (7, 9, 47, 48, 50, 51, 85–89). Clinically conspicuous humans exhibit unspecific signs like undulant fever, fatigue, weakness, lymphadenopathy, liver and spleen enlargement (7, 44, 47, 49, 50, 90).

In summary, reasons for the occurrence of *B. canis* in Europe could be poor health in selected breeding kennels, the rising international dog trade of breeding animals (5, 19) and presence of stray dog populations (6) in some Southern and Eastern European countries (91, 92). Breeding for commercial purposes in very poor housing conditions without veterinary care may constitute additional risks. Import of those puppies could promote transboundary carry-over of *B. canis* infections in dog populations (84, 93–96).

## Conclusion

In conclusion, infection with *Brucella canis* needs to be considered in dogs in Europe and diagnostics for *Brucella canis* infection appear indicated in young dogs with discospondylitis. PCR for rapid diagnosis of *Brucella* infection may be included in the routine work-up of dogs with discospondylitis.

## Ethics Statement

The ethics committee of the Faculty of Veterinary Medicine at LMU Munich approved the study (114-16-02-2018).

## Author Contributions

GB, AF, and KH designed and coordinated the study. FP provided the laboratory data. GB, AF, and KH determined the evaluation of the clinical and laboratory data. WH designed the antibody testing. GW designed the microbiological culture of clinical cases. FM designed the PCR examinations of clinical cases. GB, AF, KH, and WH wrote the manuscript. All authors read and approved the final manuscript.

### Conflict of Interest Statement

FP was employed by company IDEXX Laboratories (Ludwigsburg, Germany).

The remaining authors declare that the research was conducted in the absence of any commercial or financial relationships that could be construed as a potential conflict of interest.

## Supplementary Material

Supplementary Table S1 Laboratory data: PCR and serology 2014-2016.

Supplementary Table S2 Laboratory data: PCR 2011-2013.

Supplementary Table S3 Laboratory data: Serology 2011-2013.

Supplementary Table S4 Clinical cases - signalement, country of origin and summary of diagnostics and treatments.
